# *catena*-Poly[[[triaqua­copper(II)]-μ-2,2′-bipyridine-3,3′-dicarboxyl­ato-κ^3^ *N*,*N*′:*O*] monohydrate]

**DOI:** 10.1107/S1600536811046423

**Published:** 2011-11-09

**Authors:** Qihui Wu, Fuxiang Wang, Nanqing Jiang, Li Cao, Qinhe Pan

**Affiliations:** a Department of Materials and Chemical Engineering, Ministry of Education Key Laboratory of Application Technology of Hainan Superior Resources Chemical Materials, Hainan University, Haikou 570228, Hainan Province, People’s Republic of China

## Abstract

The title compound, {[Cu(C_12_H_6_N_2_O_4_)(H_2_O)_3_]·H_2_O}_*n*_, was synthesized under hydro­thermal conditions. The Cu^2+^ ion is six-coordinated by three water O atoms, and two N atoms and one O atom of the 2,2′-bipyridine-3,3′-dicarboxyl­ate bridging ligand in a sligthly distorted octa­hedral environment. The 2,2-bipyridine-3,3′-dicarboxyl­ate bridges link the Cu^2+^ ions into chains along the *b*-axis direction. These chains are further linked by O—H⋯O hydrogen bonds involving the water solvent mol­ecules, forming a three-dimensional framework.

## Related literature

For potential applications of coordination polymers in drug delivery, shape-selective sorption/separation and catalysis, see: Chen & Tong (2007 ▶); Zeng *et al.* (2009 ▶). Their structures vary from one-dimensional to three-dimensional architectures, see: Du & Bu (2009 ▶); Qiu & Zhu (2009 ▶). For our recent research on the synthesis of coordination polymers, see: Pan *et al.* (2010*a* ▶,*b* ▶,*c* ▶, 2011 ▶). 
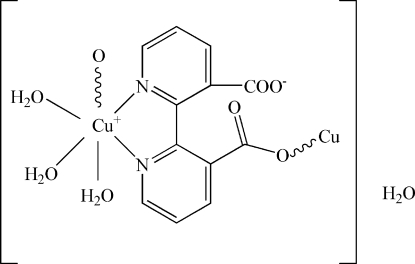


## Experimental

### 

#### Crystal data

[Cu(C_12_H_6_N_2_O_4_)(H_2_O)_3_]·H_2_O
*M* *_r_* = 377.79
Monoclinic, 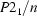

*a* = 9.950 (4) Å
*b* = 9.161 (4) Å
*c* = 15.974 (7) Å
β = 96.848 (8)°
*V* = 1445.7 (10) Å^3^
*Z* = 4
Mo *K*α radiation
μ = 1.56 mm^−1^
*T* = 296 K
0.30 × 0.18 × 0.15 mm

#### Data collection

Bruker APEXII CCD area-detector diffractometer
Absorption correction: multi-scan (*SADABS*; Bruker, 2005 ▶) *T*_min_ = 0.722, *T*_max_ = 0.792
10263 measured reflections
3585 independent reflections
2268 reflections with *I* > 2σ(*I*)
*R*_int_ = 0.062

#### Refinement

*R*[*F* ^2^ > 2σ(*F* ^2^)] = 0.054
*wR*(*F* ^2^) = 0.153
*S* = 1.08
3585 reflections
208 parameters
H-atom parameters constrained
Δρ_max_ = 1.02 e Å^−3^
Δρ_min_ = −1.13 e Å^−3^

### 

Data collection: *APEX2* (Bruker, 2005 ▶); cell refinement: *SAINT* (Bruker, 2005 ▶); data reduction: *SAINT*; program(s) used to solve structure: *SHELXS97* (Sheldrick, 2008 ▶); program(s) used to refine structure: *SHELXL97* (Sheldrick, 2008 ▶); molecular graphics: *SHELXTL* (Sheldrick, 2008 ▶); software used to prepare material for publication: *SHELXTL*.

## Supplementary Material

Crystal structure: contains datablock(s) I, global. DOI: 10.1107/S1600536811046423/yk2027sup1.cif

Structure factors: contains datablock(s) I. DOI: 10.1107/S1600536811046423/yk2027Isup2.hkl

Additional supplementary materials:  crystallographic information; 3D view; checkCIF report

## Figures and Tables

**Table 1:** Hydrogen-bond geometry (Å, °)

*D*—H⋯*A*	*D*—H	H⋯*A*	*D*⋯*A*	*D*—H⋯*A*
O5—H5*A*⋯O1*W*^i^	0.85	1.84	2.669 (5)	167
O5—H5⋯O4^ii^	0.85	1.86	2.689 (4)	167
O6—H6*A*⋯O3^iii^	0.85	1.88	2.715 (5)	169
O6—H6⋯O1^i^	0.85	2.43	3.282 (5)	180
O7—H7*A*⋯O4^i^	0.85	1.80	2.642 (4)	170
O7—H7⋯O1^ii^	0.85	1.95	2.711 (4)	149
O1*W*—H1*WA*⋯O1	0.85	2.11	2.850 (5)	146
O1*W*—H1*W*⋯O3	0.85	2.01	2.854 (6)	170

Symmetry codes: (i) 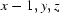; (ii) 
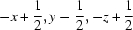
; (iii) 
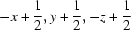
.

**Fig. 1.**
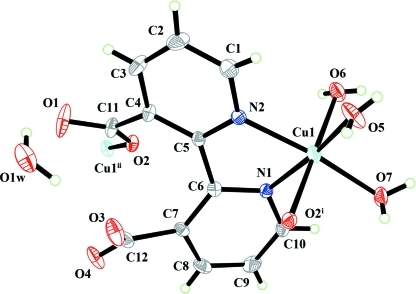
A view of the structure of complex. Ellipsoids are drawn at the 30% probability level. [Symmetry codes: (i) 1/2 - x, -1/2 + y, 1/2 - z; (ii) 1/2 - x, 1/2 - y, 1/2 - z.]

## supplementary crystallographic information

### Comment

The design and synthesis of coordination polymers have attracted increasing attention in recent years because of their potential applications in drug delivery, shape-selective sorption/separation, and catalysis (Chen *et al.*, 2007 and Zeng *et al.*, 2009). Their structures vary from one-dimensional to three-dimensional architectures (Qiu *et al.*, 2009 and Du *et al.*, 2009). In our recent works, our research interest has been focused on the synthesis of coordination polymers (Pan *et al.*, 2010*a*,*b*,*c* and 2011). Here we present a Cu-containing coordination polymer with one-dimensional chain structure.

As shown in Fig. 1, the asymmetric part of crystal structure of the title compound consists of an Cu atoms, a 2,2'-bipyridine-3,3'-dicarboxylate (bpdc) unit, three coordinated water molecules and one solvate water molecules. The Cu center is six-coordinated by four O atoms and two N atoms. Three of the four O atoms are from three coordination water molecules and the last one is from the carboxylate of the bpdc unit, whereas both N atoms are from the bridging bpdc unit. By this way, the Cu centers and the bpdc units form a chain-like structure, and these chains are further linked by hrydrogen bonds involving the solvent water molecules to from a three-dimensional superamolecular framework (see Table 1).

### Experimental

In a typical synthesis, a mixture of CuSO_4_ (0.032 g), bpdc (0.026 g), NaOH (0.008 g), 2,2'-bipyridine (0.016 g) and H_2_O (10 ml), was placed into a 25 ml Teflon-lined reactor under autogenous pressure at 100 °C for 3 days.

### Refinement

All H atoms were positioned geometrically (C—H = 0.93 Å and O—H = 0.85 Å) and allowed to ride on their parent atoms with *U*ĩso~(H) = 1.2*U*_eq_(parent atom).

### Crystal data

**Table:**

[Cu(C_12_H_6_N_2_O_4_)(H_2_O)_3_]·H_2_O	*Z* = 4
*M**_r_* = 377.79	*F*(000) = 772
Monoclinic, *P*2_1_/*n*	*D*_x_ = 1.736 Mg m^−^^3^
Hall symbol: -P 2yn	Mo *K*α radiation, λ = 0.71073 Å
*a* = 9.950 (4) Å	θ = 1.0–28.4°
*b* = 9.161 (4) Å	µ = 1.56 mm^−^^1^
*c* = 15.974 (7) Å	*T* = 296 K
β = 96.848 (8)°	Rod-like, blue
*V* = 1445.7 (10) Å^3^	0.3 × 0.18 × 0.15 mm

### Data collection

**Table:**

Bruker APEXII CCD area-detector diffractometer	3585 independent reflections
Radiation source: fine-focus sealed tube	2268 reflections with *I* > 2σ(*I*)
graphite	*R*_int_ = 0.062
Detector resolution: 5.00 pixels mm^-1^	θ_max_ = 28.4°, θ_min_ = 2.3°
phi and ω scans	*h* = −10→13
Absorption correction: multi-scan (*SADABS*; Bruker, 2005)	*k* = −12→12
*T*_min_ = 0.722, *T*_max_ = 0.792	*l* = −21→21
10263 measured reflections	

### Refinement

**Table:**

Refinement on *F*^2^	Primary atom site location: structure-invariant direct methods
Least-squares matrix: full	Secondary atom site location: difference Fourier map
*R*[*F*^2^ > 2σ(*F*^2^)] = 0.054	Hydrogen site location: inferred from neighbouring sites
*wR*(*F*^2^) = 0.153	H-atom parameters constrained
*S* = 1.08	*w* = 1/[σ^2^(*F*_o_^2^) + (0.0663*P*)^2^ + 0.4177*P*] where *P* = (*F*_o_^2^ + 2*F*_c_^2^)/3
3585 reflections	(Δ/σ)_max_ < 0.001
208 parameters	Δρ_max_ = 1.02 e Å^−^^3^
0 restraints	Δρ_min_ = −1.13 e Å^−^^3^

### Special details

**Table:**

Geometry. All s.u.'s (except the s.u. in the dihedral angle between two l.s. planes) are estimated using the full covariance matrix. The cell s.u.'s are taken into account individually in the estimation of s.u.'s in distances, angles and torsion angles; correlations between s.u.'s in cell parameters are only used when they are defined by crystal symmetry. An approximate (isotropic) treatment of cell s.u.'s is used for estimating s.u.'s involving l.s. planes.
Refinement. Refinement of *F*^2^ against ALL reflections. The weighted *R*-factor *wR* and goodness of fit *S* are based on *F*^2^, conventional *R*-factors *R* are based on *F*, with *F* set to zero for negative *F*^2^. The threshold expression of *F*^2^ > σ(*F*^2^) is used only for calculating *R*-factors(gt) *etc*. and is not relevant to the choice of reflections for refinement. *R*-factors based on *F*^2^ are statistically about twice as large as those based on *F*, and *R*- factors based on ALL data will be even larger.

### Fractional atomic coordinates and isotropic or equivalent isotropic displacement parameters (Å^2^)

**Table:**

	*x*	*y*	*z*	*U*_iso_*/*U*_eq_	
Cu1	−0.03139 (5)	0.32126 (5)	0.24233 (3)	0.02945 (18)	
O1	0.5391 (3)	0.5386 (4)	0.1205 (3)	0.0636 (12)	
O2	0.4238 (3)	0.6377 (3)	0.21712 (17)	0.0274 (6)	
O3	0.4864 (3)	0.2420 (4)	0.2535 (2)	0.0508 (9)	
O4	0.6054 (3)	0.4033 (3)	0.3363 (2)	0.0453 (9)	
O5	−0.1561 (3)	0.1917 (3)	0.1632 (2)	0.0487 (9)	
H5A	−0.2390	0.2119	0.1489	0.058*	
H5	−0.1267	0.1045	0.1642	0.058*	
O6	−0.1426 (3)	0.5059 (3)	0.19990 (19)	0.0373 (7)	
H6A	−0.1026	0.5863	0.2126	0.045*	
H6	−0.2252	0.5142	0.1795	0.045*	
O7	−0.1432 (3)	0.3074 (3)	0.3421 (2)	0.0375 (7)	
H7A	−0.2269	0.3278	0.3398	0.045*	
H7	−0.1294	0.2276	0.3689	0.045*	
N1	0.1256 (3)	0.4331 (3)	0.31322 (19)	0.0232 (7)	
N2	0.1029 (3)	0.3501 (3)	0.1547 (2)	0.0243 (7)	
C1	0.0730 (4)	0.3334 (5)	0.0718 (3)	0.0346 (10)	
H1	−0.0109	0.2945	0.0514	0.042*	
C2	0.1597 (5)	0.3706 (6)	0.0152 (3)	0.0438 (12)	
H2	0.1373	0.3541	−0.0422	0.053*	
C3	0.2821 (5)	0.4335 (5)	0.0460 (3)	0.0399 (11)	
H3	0.3430	0.4599	0.0088	0.048*	
C4	0.3149 (4)	0.4575 (4)	0.1313 (2)	0.0264 (8)	
C5	0.2231 (4)	0.4074 (4)	0.1854 (2)	0.0225 (8)	
C6	0.2437 (4)	0.4159 (4)	0.2795 (2)	0.0230 (8)	
C7	0.3662 (4)	0.4059 (4)	0.3308 (2)	0.0261 (9)	
C8	0.3664 (4)	0.4325 (5)	0.4164 (3)	0.0370 (10)	
H8	0.4477	0.4297	0.4517	0.044*	
C9	0.2479 (5)	0.4629 (6)	0.4497 (3)	0.0420 (11)	
H9	0.2483	0.4865	0.5063	0.050*	
C10	0.1292 (4)	0.4572 (5)	0.3962 (3)	0.0336 (10)	
H10	0.0479	0.4707	0.4186	0.040*	
C11	0.4367 (4)	0.5507 (5)	0.1593 (3)	0.0335 (10)	
C12	0.4963 (4)	0.3469 (4)	0.3029 (3)	0.0304 (9)	
O1W	0.5895 (3)	0.2368 (4)	0.0943 (3)	0.0610 (11)	
H1WA	0.5835	0.3265	0.0808	0.073*	
H1W	0.5492	0.2392	0.1383	0.073*	

### Atomic displacement parameters (Å^2^)

**Table:**

	*U*^11^	*U*^22^	*U*^33^	*U*^12^	*U*^13^	*U*^23^
Cu1	0.0221 (3)	0.0281 (3)	0.0382 (3)	−0.0002 (2)	0.0039 (2)	0.0025 (2)
O1	0.054 (2)	0.065 (2)	0.082 (3)	−0.0323 (19)	0.051 (2)	−0.039 (2)
O2	0.0259 (14)	0.0270 (14)	0.0306 (15)	−0.0058 (12)	0.0086 (11)	−0.0046 (12)
O3	0.0329 (17)	0.044 (2)	0.076 (3)	0.0054 (15)	0.0063 (17)	−0.0190 (19)
O4	0.0202 (15)	0.0339 (17)	0.079 (3)	0.0038 (13)	−0.0036 (15)	−0.0010 (17)
O5	0.0301 (17)	0.0296 (17)	0.081 (3)	−0.0008 (13)	−0.0178 (16)	−0.0044 (16)
O6	0.0261 (15)	0.0325 (16)	0.0517 (19)	0.0042 (12)	−0.0018 (13)	0.0067 (14)
O7	0.0219 (14)	0.0347 (17)	0.058 (2)	0.0061 (12)	0.0150 (13)	0.0121 (14)
N1	0.0223 (16)	0.0232 (16)	0.0243 (17)	0.0012 (13)	0.0033 (13)	0.0001 (13)
N2	0.0232 (16)	0.0259 (17)	0.0237 (17)	−0.0036 (13)	0.0018 (13)	−0.0011 (13)
C1	0.034 (2)	0.038 (2)	0.031 (2)	−0.0079 (19)	0.0003 (18)	−0.0036 (19)
C2	0.057 (3)	0.049 (3)	0.026 (2)	−0.006 (2)	0.002 (2)	−0.006 (2)
C3	0.049 (3)	0.043 (3)	0.030 (2)	−0.008 (2)	0.016 (2)	−0.003 (2)
C4	0.027 (2)	0.026 (2)	0.028 (2)	−0.0050 (16)	0.0089 (16)	−0.0048 (16)
C5	0.0226 (18)	0.0193 (18)	0.026 (2)	0.0027 (15)	0.0040 (15)	−0.0014 (15)
C6	0.0201 (18)	0.0187 (18)	0.030 (2)	−0.0027 (15)	0.0026 (15)	−0.0003 (15)
C7	0.0236 (19)	0.0221 (19)	0.031 (2)	−0.0005 (15)	−0.0016 (16)	−0.0004 (16)
C8	0.033 (2)	0.040 (3)	0.034 (2)	−0.003 (2)	−0.0097 (19)	−0.001 (2)
C9	0.045 (3)	0.060 (3)	0.021 (2)	−0.007 (2)	0.0029 (19)	−0.003 (2)
C10	0.034 (2)	0.038 (2)	0.031 (2)	−0.0007 (19)	0.0101 (18)	−0.0053 (19)
C11	0.030 (2)	0.032 (2)	0.040 (3)	−0.0075 (18)	0.0146 (18)	−0.0015 (19)
C12	0.0213 (19)	0.025 (2)	0.044 (3)	0.0033 (16)	0.0005 (17)	0.0032 (18)
O1W	0.040 (2)	0.056 (2)	0.086 (3)	0.0137 (18)	0.0043 (19)	−0.014 (2)

### Geometric parameters (Å, °)

**Table:**

Cu1—O5	2.043 (3)	N2—C5	1.344 (5)
Cu1—O7	2.053 (3)	C1—C2	1.365 (6)
Cu1—O2^i^	2.056 (3)	C1—H1	0.9300
Cu1—N2	2.064 (3)	C2—C3	1.383 (6)
Cu1—N1	2.085 (3)	C2—H2	0.9300
Cu1—O6	2.090 (3)	C3—C4	1.380 (6)
O1—C11	1.259 (5)	C3—H3	0.9300
O2—C11	1.239 (5)	C4—C5	1.408 (5)
O2—Cu1^ii^	2.056 (3)	C4—C11	1.506 (5)
O3—C12	1.241 (5)	C5—C6	1.495 (5)
O4—C12	1.261 (5)	C6—C7	1.388 (5)
O5—H5A	0.8500	C7—C8	1.389 (6)
O5—H5	0.8500	C7—C12	1.518 (5)
O6—H6A	0.8501	C8—C9	1.379 (6)
O6—H6	0.8509	C8—H8	0.9300
O7—H7A	0.8500	C9—C10	1.373 (6)
O7—H7	0.8499	C9—H9	0.9300
N1—C10	1.341 (5)	C10—H10	0.9300
N1—C6	1.359 (5)	O1W—H1WA	0.8500
N2—C1	1.332 (5)	O1W—H1W	0.8500
			
O5—Cu1—O7	95.69 (14)	C1—C2—C3	117.9 (4)
O5—Cu1—O2^i^	88.56 (12)	C1—C2—H2	121.1
O7—Cu1—O2^i^	90.86 (11)	C3—C2—H2	121.1
O5—Cu1—N2	92.82 (14)	C4—C3—C2	120.8 (4)
O7—Cu1—N2	171.37 (12)	C4—C3—H3	119.6
O2^i^—Cu1—N2	87.91 (12)	C2—C3—H3	119.6
O5—Cu1—N1	169.05 (13)	C3—C4—C5	117.4 (4)
O7—Cu1—N1	92.80 (12)	C3—C4—C11	118.1 (4)
O2^i^—Cu1—N1	84.41 (12)	C5—C4—C11	124.1 (3)
N2—Cu1—N1	78.58 (12)	N2—C5—C4	121.2 (3)
O5—Cu1—O6	90.61 (12)	N2—C5—C6	113.4 (3)
O7—Cu1—O6	89.24 (11)	C4—C5—C6	125.4 (3)
O2^i^—Cu1—O6	179.16 (12)	N1—C6—C7	121.0 (4)
N2—Cu1—O6	92.12 (12)	N1—C6—C5	112.5 (3)
N1—Cu1—O6	96.42 (12)	C7—C6—C5	126.5 (3)
C11—O2—Cu1^ii^	131.7 (3)	C6—C7—C8	117.8 (4)
Cu1—O5—H5A	122.6	C6—C7—C12	124.8 (4)
Cu1—O5—H5	110.6	C8—C7—C12	116.7 (4)
H5A—O5—H5	122.0	C9—C8—C7	121.0 (4)
Cu1—O6—H6A	114.2	C9—C8—H8	119.5
Cu1—O6—H6	130.3	C7—C8—H8	119.5
H6A—O6—H6	114.7	C10—C9—C8	117.7 (4)
Cu1—O7—H7A	124.9	C10—C9—H9	121.2
Cu1—O7—H7	111.9	C8—C9—H9	121.2
H7A—O7—H7	108.0	N1—C10—C9	122.8 (4)
C10—N1—C6	119.2 (3)	N1—C10—H10	118.6
C10—N1—Cu1	123.3 (3)	C9—C10—H10	118.6
C6—N1—Cu1	110.8 (2)	O2—C11—O1	125.8 (4)
C1—N2—C5	119.4 (3)	O2—C11—C4	115.8 (3)
C1—N2—Cu1	125.1 (3)	O1—C11—C4	118.3 (4)
C5—N2—Cu1	115.0 (2)	O3—C12—O4	125.9 (4)
N2—C1—C2	123.1 (4)	O3—C12—C7	117.1 (4)
N2—C1—H1	118.4	O4—C12—C7	116.8 (4)
C2—C1—H1	118.4	H1WA—O1W—H1W	99.2

Symmetry codes: (i) −*x*+1/2, *y*−1/2, −*z*+1/2; (ii) −*x*+1/2, *y*+1/2, −*z*+1/2.

### Hydrogen-bond geometry (Å, °)

**Table:**

*D*—H···*A*	*D*—H	H···*A*	*D*···*A*	*D*—H···*A*
O5—H5A···O1W^iii^	0.85	1.84	2.669 (5)	167.
O5—H5···O4^i^	0.85	1.86	2.689 (4)	167.
O6—H6A···O3^ii^	0.85	1.88	2.715 (5)	169.
O6—H6···O1^iii^	0.85	2.43	3.282 (5)	180.
O7—H7A···O4^iii^	0.85	1.80	2.642 (4)	170.
O7—H7···O1^i^	0.85	1.95	2.711 (4)	149.
O1W—H1WA···O1	0.85	2.11	2.850 (5)	146.
O1W—H1W···O3	0.85	2.01	2.854 (6)	170.

Symmetry codes: (iii) *x*−1, *y*, *z*; (i) −*x*+1/2, *y*−1/2, −*z*+1/2; (ii) −*x*+1/2, *y*+1/2, −*z*+1/2.
